# Isolation, characterization and prevention of various microbial strains in NIC unit and PIC unit

**DOI:** 10.1038/s41598-020-79364-1

**Published:** 2021-01-12

**Authors:** M. Amin Mir, Muhammad Waqar Ashraf, Vibha Tripathi, Bilal Ahmad Mir

**Affiliations:** 1 Department of Mathematics and Natural Sciences, Prince Mohammad Bin Fahd University, Al Khobar, Saudi Arabia; 2 Uttaranchal (PG) College of Biomedical Sciences and Hospital Dehradun, Sewla Khurd, Uttarakhand India

**Keywords:** Biochemistry, Immunology, Microbiology

## Abstract

The health of the hospital associated persons, particularly those dealing directly with insertion of devices, are serious cause of concern for hospitals. In this study, the most prevalent organism on the surface of medical devices in PICU were *CoNS* (16.66%) and *Staphylococcus aureus* (16.66%), while in NICU the most prevalent organism was *Klebsiella *spp. (11.25%) among *Entero-bacteriaceae* group followed by *Acinetobacter baumannii* (10%),* Escherichia coli* (2.5%), *CoNS* (6.25%), *S. aureus* (6.25%) and *Enterococcus faecalis* (6.25%). The most common species identified from blood specimen of clinical samples shows the maximum presence of *Candida *sp. (60/135) followed by *A. baumannii* (21/135), *Klebsiella Pneumoniae* (20/135), *Enterococci* (12/135), Burkholderia *cepacia complex* (8/135), *S. aureus* (6/135), *E. coli* (5/135), *Pseudomonas aeruginosa* (3/135). Different antibiotics have been used against these micro-organisms; but Cotrimoxazole, Vancomycin have been found more effective against *CoNS bacteria*, Clindamycin, Tetracycline for *S. aureus*, Nitofurantoin for Acinetobacter, and for *E. faecalis*, *A. baumanii*, and *Klebsiella*, erythromycin, Colistin, and Ceftriaxone have been found more effective respectively.

## Introduction

The infections (HCAIs/HAIs) of the health care associates are indicators of the out comings of poor quality of patient care. The Infections of the health-care set-ups have direct adverse Consequence, which affect the patients, their families, visitors and society as well. The control of infection of HAIs is therefore the need of an hour. Thus the growing concern about HAIs, together with patient’s safety leads the World Health Organization (WHO) to make the World Alliance Safety. An initiative in terms of prevention of HAIs was Alliance’s First Global Patient Safety plan, in terms of ‘Clean Care is Safer Care^1^. The Control and preventive activities can only be achieved by sound and systematic base-line data. So the surveillance of HAI has become a common and important set up in many countries^2^. Surveillance systems are therefore plays an important role in designing evidences for the prevention and control of numerous infections.

As the traditional manual methods of surveillance methods are less efficient because of more time Consumption, limited in scope, insensitive, often applied in consistently^3^ should be over combed. In most of the hospital surveillance activities are passive, involving data analysis of microbiological culture reports of routinely sent diagnostic samples. There is a difference in the infection rate of adults, young and infants, because of difference in their immune systems. These differences occur due to differential use of mechanical devices and surgical procedures in this population. *Escherichia coli* the most common pathogen in adults (14% of all nosocomial infections), also in pediatrics patients, *Staphylococcus aureus* has been found the most common isolate 31%^4,5^.

### Study area and study period

The present experimentation was performed in the Microbiology Department, Uttaranchal College of Biomedical Sciences and Hospital, Dehradun. Samples were collected from NICU & PICU of Shri Mahant Indiresh Hospital, Dehradun from September 2019 to February 2020. All methods were carried out in accordance with relevant guidelines and regulations. (Declaration of Helsinki). Also the study protocol was approved by ethic committee of Shri Mahant Indiresh Hospital, Dehradun. The written informed Consent was obtained from all the participants.

### Specimen collection

Specimens were collected from the surface of the instruments using moisten (0.9% w/v physiological saline) sterile cotton swab. Similarly, nasal and hand swabs were taken.

### Sample size and sample technique

A total of 260 samples were Considered for bacterial examination, 80 from different equipment’s, 30 nasal swabs and 30 hand swabs from NICU and 60 from different equipment’s 30 nasal swabs and 30 hand swabs from PICU.

### Identification

Identification of organism was done as follows:

### Gram stain^6^

Gram stain is an extensively used differentiation method that splits bacteria into two major groups; those who retain crystal violet dye after treatment with iodine and alcohol/acetone appear purple are referred as gram positive and the bacteria which losses the crystal violet and shows the counter stain colour is labeled as gram negative.

#### Procedure

A smear was made on a clean glass slide, air dried and fixed by flame of a burner.

Crystal violet—1 min → Washed the slide with water → Grams iodine—1 min → Washed the slide with water → Decolorized with acetone –2–3 s → Washed the slide with water immediately → Safranine—30 s → Washed with water, air dried and examined under 100X oil.

### Motility by hanging drop preparation^7^

#### Procedure

A hollow ground slide i.e. with a shallow, circular concavity in it centre were used. Encircle the concavity with a line streak of soft petroleum jelly applied with a glass rod to the surface of the slide just outside the concavity. A minute drop or suspension of the liquid culture was placed on the surface of the cover slip then the slide was inverted over the cover slip, allow to adhere the jelly, which is then immediately turned so that the cover-slip is uppermost. The liquid drop will then hang from the cover slip in the center of the concavity which was observed under high power 40X.

### Catalase test^8^

#### Procedure

With a wooden applicator stick or cover slip, growth was transformed from the Centre point of the colony to the surface of glass slide. 1 ml of 3% Hydrogen peroxide was added for bubble formation.

### Coagulase test^8^

#### Bound coagulase

##### Procedure

Two drops of normal sterile saline was placed on a glass slide. Emulsified colony material from the organism to be identified is put in a liquid in the concerned circles for milky white suspension formation. Then a small amount of plasma is placed in the suspension and mix with a wooden applicator stick. Another drop of saline in the other suspension was placed as a control. Then the slide was rocked back and forth, observing for agglutination of the test suspension.

#### Free coagulase

##### Procedure

A little quantity of colony growth of the organisms was emulsified with 0.5 ml of plasma (1:6 diluted) in a small tube and were then incubated at 38 °C for 4 h. and observed for clot formation by gently tilting the tube. In case of no clot formation, the tubes are re-incubated at room temperature and read again after 18 h.

#### Nutrient agar^6^

##### Procedure

Near about 28 g of dehydrated powder was mixed with 1000 ml of distilled water, heated to dissolve the medium completely. It was sterilized, autoclaved at about 15lbs pressure at 121 °C for 15 min, and finally the contents were cooled to 45–50 °C and then poured into sterile petri plates.

#### Blood agar^6^

##### Procedure

The medium was prepared by adding sterile 5–10% blood to sterile nutrient agar that has been melted and cooled to 50 °C and then poured into Petri plates.

#### Macconkey’s agar^6^

##### Procedure

55.04 g was dissolved in 1000 ml of distilled water, heated to complete uniformity of the mixture and then completely sterilized by autoclaving at 15 lbs. pressure 121 °C for 15 min. Cooled to 45–50 °C and poured into sterile Petri plates.

#### Mannitol salt agar^6^

##### Procedure

111.02 g was mixed in 1000 ml of purified distilled water, heated until boiled for complete dissolutions within the medium, and then sterilized by autoclaving at 15lbs pressure 121 °C for 15 min.

### Biochemical tests

#### Indole test^8^

##### Procedure

Tryptophan broth inoculated with 1 drop from a 24 h brain heart infusion, incubated at 35 °C in an ambient air for 48 h, and then 0.5 ml of Kovac’s reagent was added to the broth culture.

#### Methyl Red/Voges Proskauer test^8^

##### Procedure

MR/VP broth was inoculated with 1 drop of broth culture from a 24 h brain heart infusion culture, which was incubated at 35°–38° for a minimum of 48 h in ambient air.

#### Citrate utilization test^8^

##### Procedure

Simmons citrate agar was inoculated on slant by the tip of a needle to a colony that is 18–24 h old, then incubated at 35°–38° for up to 8 days. The mixture was observed for growth of blue color, denoting alkalinization.

#### Bile Esculin test^8^

Few colonies of culture was inoculated for about 18–24 on the surface of the slant of bile esculin agar, then again incubated at 35°–38° in an ambient air for 48 h.

#### Nitrate reduction test^6^

##### Procedure

Nitrate broth was inoculated with 1–2 drops from the young broth culture. After completion of incubation, 1 ml of each reagent A and B was added to the test medium.

#### Oxidase test^8^

##### Procedure

Filter paper was moistened with the substrate or a commercially available paper disk that has been impregnated with the substrate is used.

Using a platinum wire or wooden stick, a small portion of bacterial colony from the agar surface were removed and rubbed on the filter paper or commercial disk.

Than observed the inoculated area of paper or disk for a color changes to deep blue or purple color within 10 s.

#### Urease test^8^

##### Procedure

The surface of urea agar slant was streaked with a small portion of well isolated colony and the slant was inoculated with 1–2 drops from an overnight brain–heart infusion broth culture.

The cap was loosed and the tubes were incubated at 35°–38° in ambient air for 48 h to 8 days.

#### L-Pyrrolidonyl Arylamidase (PYR) test^8^

##### Procedure

Before inoculation, the disk was moistened lightly with Analytical grade water. A wooden applicator stick was used to rub a small amount of several colonies for about 18–24 h with pure culture on small area of PYR disk then incubated at room temperature for 2 min. A small portion of detector reagent N, N-dimethylamino-cinnamaldehyde was added and observed for red color development within 1 min.

#### Fermentation media^8^

##### Procedure

A tube was inoculated with 1 drop of 18–24 h brain–heart infusion broth culture and was then incubated at 35–38 °C for about 8 days. Acid and gas production is examined.

#### Decarboxylase test^8^

##### Procedure

Tubes were inoculated with a drop of brain–heart infusion broth culture, and a thin layer of sterile mineral oil was added to each tube. The cultures were incubated for 4 days at 35°–38° in ambient air and were examined at 24, 48, 82, 96 h.

#### Triple sugar iron^8^

##### Procedure

By using a straight inoculation needle, the top of a well isolated colony was touched. TSI was first inoculated by stabbing through the medium to the middle of the tube and then streaking the surface of the agar slant. The cap was loosed and the tubes were incubated at 35°-38°c in excess air for 18–24 h.

#### Deoxyribonuclease test^6^

##### Procedure

42.0 g of dehydrated media was suspended in 1000 ml in AR grade water and then heated with frequent agitation to mix the medium completely. The mixture after sterilization by autoclaving at 118°–121° for 15 min, was then made to cool to 45°, finally poured in sterile Petri plates. Then 100 mg toluidine blue was added before sterilizing the medium or the plates were flooded with 0.1% toluidine blue after incubation as desired.

#### Phenolphthalein phosphatase test^6^

##### Procedure

28.01 g of dehydrated powder was mixed in 1000 ml of distilled water and then heated to boil for complete dissolution of medium. Do not autoclave. Dispense as desired.

#### Gelatin hydrolysis test^6^

##### Procedure

34.00 g of dehydrated powder was dissolved in 1000 ml of distilled water with continuous heating to dissolve the medium completely, then autoclaved at 121 °C for 30 min in a dispensed plate.

#### Antibiotic susceptibility test^8^

Antibiotic susceptibility test was performed on the basis of organism isolated and antibiotic were selected according to CLSI guidelines. The antimicrobial susceptibility test was carried out Kirby Bauer disk diffusion method.

#### Muller Hinton agar^6^

##### Procedure

Nearly 38.0 g were dissolved in 1000 ml of distilled water, with continuous heating for the dissolution of medium completely, and then sterilization was done by autoclaving at 15lbs pressure at 121 °C for 15 min.

Muller Hinton’s agar is used for isolation of gram negative bacilli and gram positive cocci except Streptococcus species.

#### Kirby Bauer disk diffusion method^8^

##### Procedure

With a wire loop the 4 to 5 colonies of well isolated similar colonies was touched from the top of agar plate and transferred to the tube that contains 4–5 ml of suitable broth medium, followed by incubation at 35 °C until it matches the 0.5 Mc Farland usually 2–6 h.

A sterile nontoxic cotton swab was put into the inoculum after it adjusts its turbidity of that of standard and the swab was made to rotate many times with continuous pressure on the inside wall of the tube in order to remove excess fluid.

Then on the dried surface of Muller Hinton’s agar plate, the swab was inoculated to bring the swab to room temperature by rotating the entire agar surface to near about 60 degrees each time and finally swab was rimmed of the agar. Finally the dish lid was replaced and allowed to dry on the surface of the agar before adding the antibiotic disk.

### Observations

In the study period of about 6 months a total of 260 samples were collected from instruments, hand and nasal from neonatal and pediatrics ICUs. Total no of samples collected from instruments in PICU is 60 and 30 each from nasal and hand (Tables 1, 2 and 3). In NICU the samples collected from instruments were 80 and 30 from hand and nasal each (Tables 4, 5 and 6).

**Table 1:** Spectrum of micro-organism isolated from medical devices associated infection in PICU.

N = 60 (PICU)	*CONS*	*S. aureus*	*A. baumanii*	*Candida *spp.	*Micrococcus*	*Bacillus*	No growth
Bed (n = 8)	2	1	–	1	–	2	2
Monitor (n = 6)	3	1	2	–	1	1	2
Infusion pump (n = 6)	–	–	–	–	–	–	0
Radiant heat warmer (n = 8)	–	2	–	–	1	–	5
Weighing machine (n = 6)	2	1	2	–	–	–	1
IV stand (n = 6)	–	2	–	–	2	–	2
X ray machine (n = 6)	1	2	–	–	–	–	3
Tubings (n = 8)	1	–	1	1	2	–	2
Ventillator (n = 8)	1	1	1	–	–	2	2
Total (%)	10 (16.6%)	10 (16.66%)	6 (10%)	2 (3.33%)	6 (10%)	5 (8.33%)	21 (35%)

Different organisms isolated from different instruments of PICU. The organisms which were isolated are *CoNS *(10), *S.aureus* (10), *Acinetobacter baumanii* (6), *candida *spp. (2), *Micrococcus* (6), *Bacillus* (5).

**Table 2:** Hand Carriage of micro-organisms in Health care workers of PICU.

Hand swab	CONS	*S. aureus*	Micrococcus	Bacillus	No growth
(n = 30)	12	6	2	4	6
	(MRCoNS = 3) 40%	(MRSA = 1) 20%	6.66%	13.3%	20%

Microorganism isolated from hands of health care workers. The organisms which were isolated are *CoNS* (12/30), *S.aureus* (6/30), *Micrococcus (*2/30), and *Bacillus* (4/30).

**Table 3:** Nasal Carriage of micro-organism in Health care workers of PICU.

Nasal wab	CONS	S. aureus	Micrococcus	No growth
(N = 30)	15	8	5	2
	(MRCoNS = 3) 50%	(MRSA = 3) 26.66%	16.66%	6.66%

Micro-organism isolated from anterior nares of health care workers. The organisms which were isolated are *CoNS* (15/30), *S.aureus* (8/30), *and Micrococcus* (5/30).

**Table 4:** Organism isolated from various site of medical instruments in NICU.

Total no of samples (n = 80)	CONS	*S. aureus*	*Klebsiella *spp.	*Acinetobacter *spp.	*E. faecalis*	*E. coli*	*Micrococcus*	No growth
Weighing machine (n = 8)	**–**	**1**	**2**	**1**	**1**	**–**	**1**	**2**
Phototherapy machine (n = 8)	**3**	**–**	**–**	**–**	**–**	**1**	**3**	**1**
Radiant heat warmer (n = 8)	**–**	**2**	**–**	**2**	**1**	**–**	**2**	**1**
Monitor (n = 8)	**–**	**–**	**–**	**–**	**–**	**–**	**1**	**8**
Oxygen hood (n = 8)	**–**	**2**	**–**	**–**	**1**	**–**	**1**	**4**
Suction machine (n = 8)	**1**	**–**	**2**	**–**	**–**	**1**	**2**	**2**
Syringe pump (n = 8)	**–**	**–**	**–**	**1**	**1**	**–**	**2**	**4**
Bed with mattress (n = 8)	**–**	**–**	**–**	**1**	**–**	**–**	**3**	**4**
Nasal cannula (n = 8)	**1**	**–**	**2**	**–**	**–**	**–**	**–**	**5**
Ventilator (n = 8)	**–**	**–**	**3**	**3**	**1**	**–**	**1**	**0**
Total	5 (6.25%)	5 (6.25%)	9 (11.25%)	8 (10%)	5 (6.25%)	2 (2.5%)	16 (20%)	30 (38.5%)

Different organisms isolated from instruments of NICU, the organisms which were isolated are *CONS* (5), *S. aureus* (5), *Klebsiella* spp. (9), *Acinetobacter *spp. (8), *Enterococcus *spp. (5), *E.coli* (5), *Micrococcus* (16).

**Table 5:** Nasal carriage of microorganism of health care workers.

Nasal swab	*CONS*	*S. aureus*	*Micrococcus*	No growth
(n = 30)	13	8	6	4
	(MRSA = 3) 43.33%	(MRSA = 2) 23.33%	20%	13.33%

Organism isolated from anteriornares of health care workers. The organism which was isolated is *CoNS* (13/30), *S.aureus* (8/30), *and Micrococcus* (6/30).

**Table 6:** Hand carriage of microorganism of health care workers.

Handswab	*CONS*	*S. aureus*	*Acinetobacter*	*K. Pneumonia*	*Bacillus*	No growth
(n = 30)	**4**	**5**	**5**	**4**	**8**	**5**
	(MRSA = 1) 13.33%	(MRSA = 2) 16.66%	16.66%	13.33%	23.33%	16.66%

Organism isolated from hands of health care workers. The organisms which were isolated are *CoNS* (4/30), *S.aureus* (5/30), *Acinetobacter* (5/30), *and K. pneumoniae* (4/30*), Bacillus* (8/30).

The samples were collected with sterile cotton tipped swabs. The swabs were moistened with sterile normal saline and streaked across a 12 mm^2^ areas and were immediately inoculated on Blood agar, MacConkey agar and the isolates were characteristics by colony identification, gram staining followed by standard biochemical reaction, and the antimicrobial activity was carried out by Kirby Bauer disk diffusion method.

## Results and discussion

**Figure 1:**
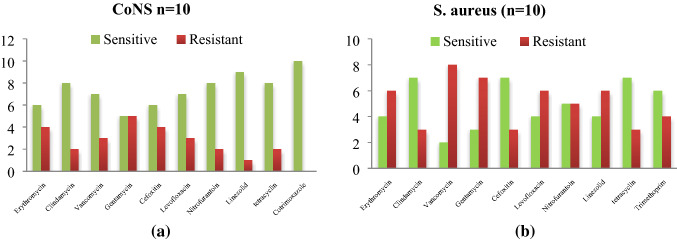
(**a**) Antibiogram of *S. aureus* isolated from instruments in PICU. (**b**) Antibiogram of *CoNS* and *S.aureus* isolated from instruments in PICU. *CoNS a*re highly sensitive to cotrimoxazole (10/10) followed by linezolid (9/12) while *S.aureus* shows a higher sensitivity to tetracycline (8/10) and vancomycin (8/10).

**Figure 2:**
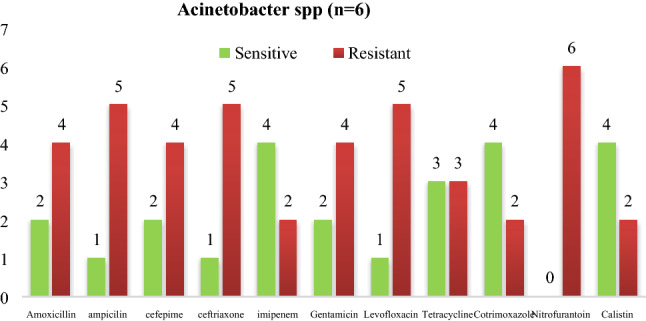
Antibiogram of *Acinetobacter *spp. isolated from instruments in PICU. *Acinetobacter *spp. were most sensitive to (4/6) colistin (4/6) followed by imipenem (4/6).

As per the antimicrobial activity is taken into Consideration *CoNS a*re highly sensitive to cotrimoxazole (10/10) followed by linezolid (9/12) while *S.aureus* shows a higher sensitivity to tetracycline (8/10) and vancomycin (8/10) as Shown in Fig. 1a,b. The *Acinetobacter *spp. were most sensitive to (4/6) colistin (4/6) followed by imipenem (4/6) as mentioned in Fig. 2.

**Figure 3:**
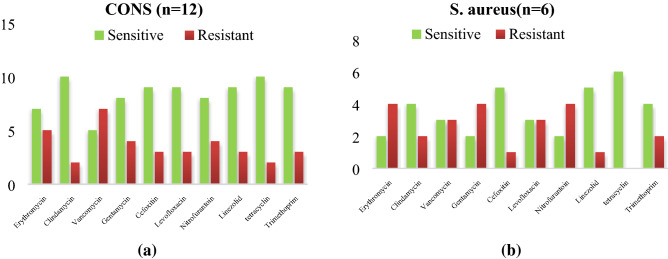
Antibiogram of CoNS and S. aureus isolated from the hands of health workers. (**a**) Antibiogram of *CoNS* isolated from hands of health care worker in PICU. (**b**) Antibiogram of *S.aureus* isolated from hands of health care worker in PICU. Out of 12 CoNS isolated, the highest sensitivity was observed for vancomycin (10/12), tetracycline (10/12), trimoxazole (9/12), while in *S.aureus* out of 6 isolates tetracycline (6/6) and linezolid (5/6) were the drugs sensitive to the isolates.

**Figure 4:**
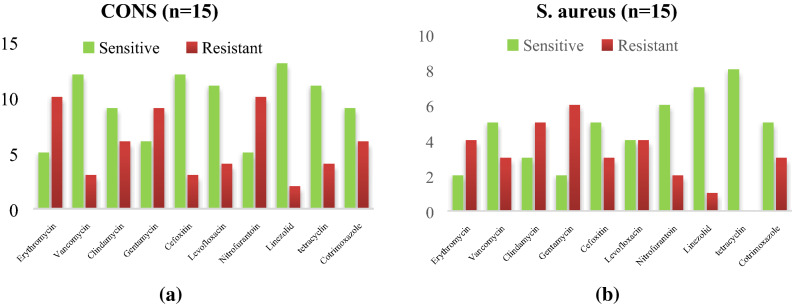
Antibiogram of *CoNS and S. aureus* Isolated from the nares of Health workers. (**a**) Antibiogram of *CoNS* isolated from anterior nares of health care worker in PICU. (**b)** Antibiogram of *S.aureus* isolated from anterior nares of health care worker in PICU. Out of 30 nasal specimen, predominant isolate was *CoNS* with a good sensitivity for linezolid (13/15), vancomycin (12/15) and MRSA were produced by 3 isolates in contrast to *S.aureus*, good sensitivity was observed for tetracycline (8/8) followed by linezolid (8/8) and Nitrofurantoin (6/8) and 3 isolates produce MRSA.

Out of 12 CoNS isolated, from the hands of health care worker in PICU the highest sensitivity was observed for vancomycin (10/12), tetracycline (10/12) and co-trimoxazole (9/12), while in *S.aureus* out of 6 isolates tetracycline (6/6) and linezolid (5/6) were the drugs sensitive to the isolates which have been found effective against the inhibition of concerned stains as depicted in Fig. 3a,b. Among the CoNS and *S.aureus* isolates from nasal specimen, linezolid (13/15), vancomycin (12/15) and tetracycline (8/8) followed by linezolid (8/8) and Nitrofurantoin (6/8) were more effective shown in the Fig. 4a,b.

**Figure 5:**
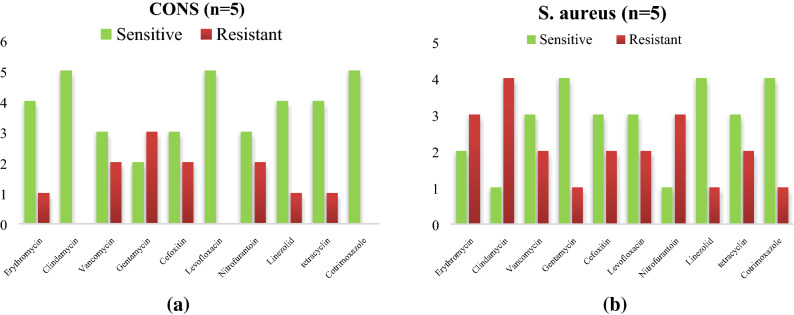
(**a**) Antibiogram of *CoNS* isolated from medical devices in NICU. (**b**) Antibiogram of *S.aureus* isolated from medical devices in NICU. Equal no of a *CoNS* and *S.aureus* isolated from NICU and both of them show a good sensitivity pattern for linezolid, co trimoxazole, vancomycin, levofloxacin and in each isolates 2 MRSA isolates were detected.

Out of 30 nasal specimen, predominant isolate was *CoNS* with a good sensitivity for linezolid (13/15), vancomycin (12/15) and MRSA were produced by 3 isolates in contrast to *S.aureus*, which show good sensitivity for tetracycline (8/8) followed by linezolid (8/8) and Nitrofurantoin (6/8) and 3 isolates produce MRSA as mentioned in Fig. 5a,b.

Surveillance of health care associates is a well-Constituted preventive measure for reduction of infections in HAIs, which are expected to be the carrier of a medium to long term infector’s systematically^8^. Nosocomial infections (NIs) are common and are increasing treat for hospitalized patients, as they are the major causes of death and disability worldwide. As per the reports of World Health Organization, up to 15% of hospitalized patients get affected by infections associated with health care workers. Also the hospitals worldwide are facing the crisis of the upsurge and dissemination of resistant bacteria, particularly associated with nosocomial infections in ICU patients^9^. Regular checkup of device-associated infections in any healthcare center becomes the fundamental issue and an informative not only to health care professionals but also to administration in making various strategies for the prevention and control of such infections^10^. The result of this study is confirming our expectation that the ICU’s are of more risk for the occurrence of nosocomial infection. In the current study as mention in Table 7 the most prevalent organism on the surface of medical devices in PICU were *CoNS* (16.66%) and *S.aureus* (16.66%) (Table 1) while in NICU the most prevalent organism was *Klebsiella *spp. (11.25%) *among* Entero-bacteriaceae group followed by *A. baumani* (*10%), E.coli*(2.5%), *CoNS* (6.25%)*, S. aureus* (6.25%) and *E. faecalis* (6.25%) (Table 4). Similar to this result, another study on environment surface of hospital wards showed that *CoNS* and *Klebsiella *spp. as predominant isolates and was the most prevalent isolated bacteria^11,12^. A study from Northwest Ethiopia also shows the *CoNS* as the most predominant pathogen^13^. However, in the study of Farzaneh Mehraban et al. showed *A. baumanii complex* and *P. aeruginosa* the most common bacteria from ICU^14–16^. A. D. Khosravi et al. have shown *Staphylococcus* as the commonest organism isolated from hospital environment^17^. Agersew Alemu et al. reported *CoNS* as the most predominant pathogen with over all isolation rates of 24.1%. Although increased rate of isolation was reported from various other studies of USA, India and Nigeria ^13^.

**Table 7:** Growth isolated from NICU and PICU.

Samples	Instruments	Health care workers
NICU	50	51
PICU	39	52
*P* value	0.8595	1.0000

*P* value > 0.05, hence the values are non-significant.

Contaminations occur either by direct transfer of contaminated health care worker or by shedding of microorganisms in the nearby vicinity of a patient’s bed^18^. In the present study among *S. Aureus* and *CoNS*, 8/30 (35%) were MRSA in PICU. Lab contaminated surfaces with resistant microorganisms’ increases the spread among patients and in the hospital environment. It is also being observed that some bacterial isolates associated with equipment’s, inanimate surfaces, the patient blood culture or other samples of patients does have similarity, and such similarities reinforces the horizontal transference of microbes^18^. As per the reports by Maya frank et al. that, no evidences were found in incline in Nosocomial bloodstream infection (NBI) in patients in Southern Israel during 1992–2001^19^. Maimon et al. mentioned an increase in the number of gram negative enteric bacterial isolates in Southern Israel in year 1989–1992 with maximum *Klebsiella *spp. (48% of all isolates) and *Enterobacter *spp. the most predominant which causes Nosocomial bloodstream infections^20^. Krontal et al. reported in year 1988–1998, that 85–90% of all *Klebsiella* spp. bacteria at PICU were identified as nosocomial. Levy et al. mentioned a nosocomial bloodstream infection rate of 48.5% during year 1988–1994 at a hospital in central Israel^21^. In this study, the frequency of *S. aureus* and among *Entero bacteriaceae*, *Klebsiella *spp. is more in contrast to study done by Vanessa Maria Sales et al. in which *Acinetobacter baumanii* was the most prevalent organism found on surface of materials and equipment^22^. Contaminated surfaces play as an edge in spreading prevalent bacteria such as *A. baumanii* and *P. aeruginosa*. It is estimated that 20 to 40% of nosocomial infection occur through the transmission of infection from the hands of hospital staff to patients^23^.

**Figure 6:**
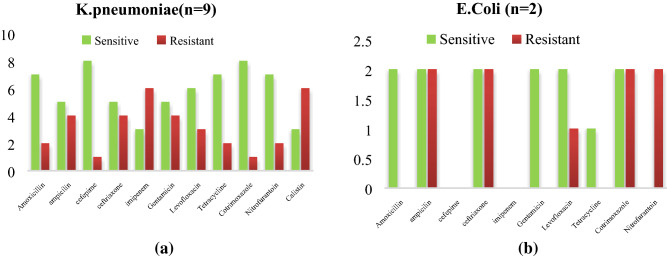
(**a**) Antibiogram of *K.pnemoniae* isolated from medical devices in NICU. (**b**) Antibiogram of *E.coli* isolated from medical devices in NICU. Among Entero-bacteriaceae, 9 *Klebsiella pneumoniae* and 2 E.coli both of them show sensitivity for colistin and imipenem.

**Figure 7:**
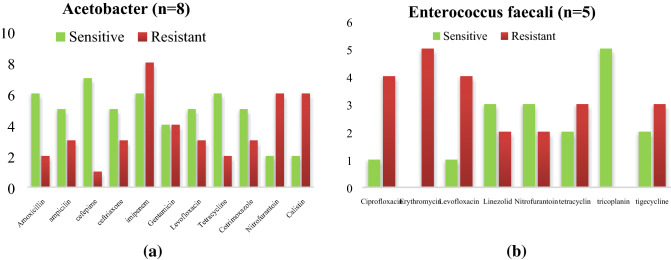
(**a**) Antibiogram of *Acinetobacter *spp. isolated from medical devices in NICU. (**b**) Antibiogram of *Enterococcus faecalis* isolated from medical devices in NICU. *Acinetobacter *spp., were least resistance to colistin (2/8) imipenem (8/8*), Enterococcus faecalis* shows more sensitivity towards Tigecycline (5/5) followed by linezolid (3/5).

**Figure 8:**
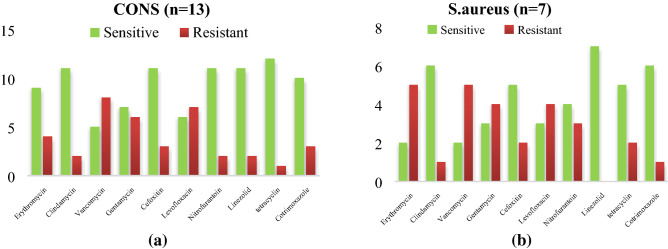
(**a**) Antibiogram of *CoNS* isolated from anterior nares of health care workers. (**b**) Antibiogram of *S.aureus isolated* from anterior nares of health care workers in NICU. *CoNS* as the predominant isolates shows a least resistant to tetracycline (1/13) and linezolid (2/13) with 3 MRSA isolated. For *S.aureus*, linezolid (8/8) susceptibility was observed with (2/8) MRSA detected.

**Figure 9:**
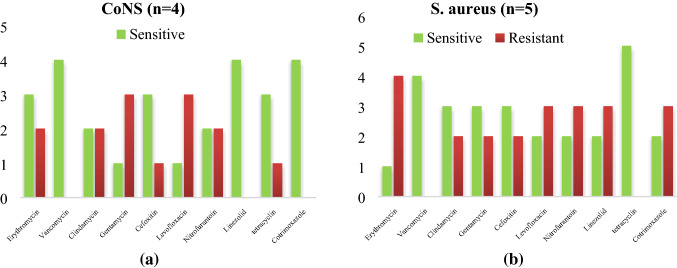
(**a**) Antibiogram of CoNS isolated from hands of health care workers of NICU. (**b**) Antibiogram of *S.aureus* isolated from hands of health care workers of NICU. Out of total *CoNS* isolated maximum sensitivity was shown for Linezolid (4/4) and Vancomycin (4/4) whereas for *S aureus* tetracycline (5/5) and Vancomycin (4/5) showing maximum sensitivity pattern.

**Figure 10:**
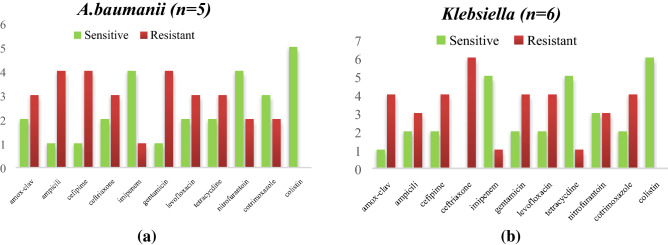
(**a**) Antibiogram *A. baumanii* isolated from hands of health care workers in NICU. (**b**) Antibiogram of *Klebsiella *spp. isolated from hands of health care workers in NICU. A good sensitivity pattern was observed for both *A. baumanii* as well as *Klebsiella *spp. for colistin (5/5, 6/6).

Equal no of a *CoNS* and *S.aureus* isolated from NICU and both of them show a good sensitivity pattern for linezolid, co trimoxazole, vancomycin, levofloxacin and in each isolates 2 MRSA isolates were detected as mentioned in Fig. 6a,b. Among Entero-bacteriaceae, 9 *Klebsiella pneumoniae* and 2 E.coli both of them show sensitivity for colistin and imipenem *Acinetobacter *spp., were least resistance to colistin (2/8) imipenem (8/8*), Enterococcus faecalis* shows more sensitivity towards Tigecycline (5/5) followed by linezolid (3/5) as mentioned in Fig. 7a,b. *CoNS* as the predominant isolates shows a least resistant to tetracycline (1/13) and linezolid (2/13) with 3 MRSA isolated. For *S.aureus*, linezolid (8/8) susceptibility was observed with (2/8) MRSA detected as mentioned in Fig. 8a,b. Out of total *CoNS* isolated maximum sensitivity was shown for Linezolid (4/4) and Vancomycin (4/4) whereas for *S aureus* tetracycline (5/5) and Vancomycin (4/5) showing maximum sensitivity pattern Fig. 9a,b. A good sensitivity pattern was observed for both *A. baumanii* as well as *Klebsiella *spp*.* for colistin (5/5, 6/6) shown in Fig. 10a,b.

**Figure 11:**
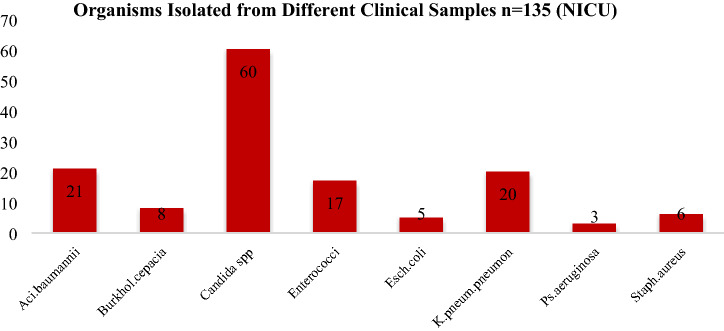
Spectrum of microorganisms isolated from clinical sample: Blood sample from NICU. Most common species identified from blood specimen of clinical samples shows the maximum presence of *Candida *spp. (60/135) followed by *Acinetobacter* baumanii (21/135), *Klebsiella Pneumoniae* (20/135)*, Enterococci* (12/135), Burkholderia *cepacia complex* (8/135), *Staphylococcus aureus* (6/135), *E.coli* (5/135), *Pseudomonas aeruginosa* (3/135).

**Figure 12:**
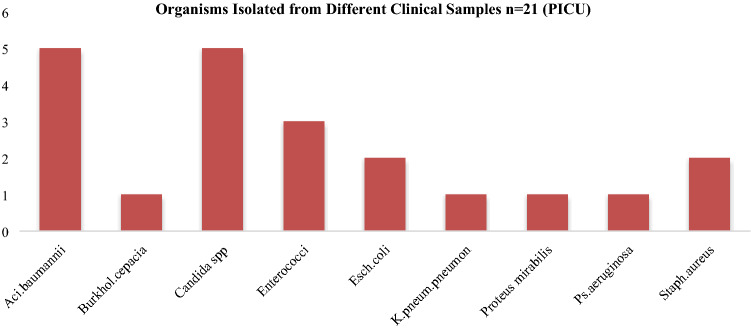
Spectrum of microorganisms isolated from clinical sample: Blood sample from PICU Isolates of blood specimen from clinical samples shows the presence of *Acinetobacter. Baumanii* (5/21) and candida spp. (5/21) in equal numbers followed by *Enterococci* (3/21), *E.coli* (2/21), *Staphylococcus aureus* (2/21), Burkholderia cepacia (1/21), *K. pneumoniae. Pneumonia* (1/21), *Proteus mirabilis* (1/21), *Pseudomonas aeruginosa* (1/21).

Antibiogram of isolates from medical devices of ICU In this study, organisms grown from medical equipments of PICU, showed CoNS and *S. aureus* chief organism, and have been found susceptible to linezolid, tetracycline and vancomycin. Highest sensitivity of *S.aureus* for vancomycin was found in studies of A. D. khosravi et al. *A. baumanii* shows susceptibility to colistin and Imipenem^24^. Similar picture has been observed by priya dutta et al. ^25^. The organisms isolated from medical equipments of NICU revealed similar picture as in PICU*. CoNS* and *S.aureus* although shows resistance to other commonly used drugs, yet linezolid, co- trimoxazole and vancomycin were found to be most effective while for *E. faecalis;* tigecycline and linezolid were sensitive (Table 4). From Entero-bacteriaceae family, colistin and imipenem has higher sensitivity pattern and same was observed for *A. baumanii*. Akash deep et al. in his study isolated 33.3% *Klebsiella *spp. and observed maximum sensitivity for amikacin^26^. Organism and the antibiogram of isolates from health care workers total of 30 health care workers each from PICU and NICU are included in the study, with a mean age of 20–30 years. Nasal and hand swabs were taken from each individual. Nasal swabs of HCWs from PICU shows a colonization of *CoNS* (15/30; Table 3), *S.aureus* (8/30; Table 3) with 6 MRSA strains detected by cefoxit in disk diffusion test. In this study the antibiotics sensitivity test revealed that *CoNS* and *S.aureus* remained sensitive to linozolid, vancomycin, tetracycline and nitro furan to in with low level of susceptibility to erythromycin, Gentamicin and Clindamycin. Ashish pathak et al. in his study isolated *S.aureus* from nasal carriage in healthy preschool children found highest sensitivity to Clindamycin^27^. At HRP Princess MCS Medical center, Thailand, *S. aureus* was found resistant to erythromycin, Clindamycin to tetracycline, chloramphenicol and fusidic acid were found resistant with the observation at the following rates: 63.2%, 63.2%, 34.2%, 2.6% and 2.6%, respectively^28^. Similar study is done for detection of microbes in hands of health care workers for which swabs from hands were taken and cultured. All participants were found to be colonized with *CoNS *(12/30; Table 2), *S.aureus* (6/30 Table 2), MRSA was found in 4 HCW (Table 2) where antibiotic susceptibility pattern revealed their maximum sensitivity for vancomycin, tetracycline, linezolid and co trimoxazole and their resistance for Clindamycin, erythromycin and nitro furan. Similarly a sum of 30 nasal and hand swabs were also collected from health care associates of NICU, which shows the presence of *CoNS* (13/30 Table 5) and *S.aureus* (8/30; Table 5) from the anterior nares and *CoNS* (4/30 Table 6), *S. aureus* (5/30 Table 6), *Acinetobacter *spp. (5/30 Table 6), *Klebsiella *spp. (4/30 Table 6), *Bacillus *spp. (8/30 Table 6) isolates from hand sampling. Antibiogram of *CoNS* depicted linezolid, vancomycin and nitrofurantoin as the most susceptible antibiotic while erythromycin and Clindamycin the most resistant one. Antibiogram from hand samples showed sensitivity of *CoNS* and *S.aureus* to linezolid, vancomycin and tetracycline and resistance for erythromycin, levofloxacin and co trimoxazole. Colistin sensitivity for *Acinetobacter *spp. and *Klebsiella *spp. remains highest. Clinical samples of blood specimen from NICU shows maximum Candida spp. (60/135), followed by *Acinetobacter baumanii* (21/135), *K. Pneumoniae* (20/135) (Fig. 11). From PICU, blood specimen from clinical samples shows the presence of *A. baumanii* (5/21) and *candida *spp. (5/21) in equal numbers followed by *Enterococci* (3/21) (Fig. 12).

Prevention is more important than management of device associated infection, simple measures of asepsis and strict adherence to hand hygiene can decrease the incidence of nosocomial infection. The continuous surveillance of hospital environment for microbial infections with continuous inspection over infection control and prevention practices will prevent HAI.

## Conclusion

A large number of factors are associated with health care associated infections in the hospitals. The Health care associated infections have significant impact on patients out comings including morbidity, mortality, hospital stay and cost of care. Therefore, a direct focus towards HCAI’s may provide a way for the health quality of health care associates and their impact on the patients. Nursing care measures have direct impact on the prevention of various types of infections, central line infections, urinary system infections, sepsis and antibiotic resistant infections. Control over patient to patient transmission of infection by using hand hygiene’s and control over general infection practices are the efficient ways for controlling the spread of resistant organisms. All persons who enter the unit should change into fresh gowns to prevent cross infection of susceptible infants. Environmental hygiene should be improved by removal of dust and dirt using vacuum cleaners with air exhaust and scrubbing of equipment’s and inanimate objects will provide a really clean environment. HEPA filter can be used for air filtration. The strict adherence to aseptic protocol must be enforced by institution and periodic surveillance of colonization of HCW as well as equipment’s should be done.
